# In-Situ Monitoring and Control of Additive Friction Stir Deposition

**DOI:** 10.3390/ma18071509

**Published:** 2025-03-27

**Authors:** Evren Yasa, Ozgur Poyraz, Khoa Do, Anthony Molyneux, James McManus, James Hughes

**Affiliations:** Advanced Manufacturing Research Centre North-West, University of Sheffield, Blackburn BB2 7HP, UK; o.poyraz@amrc.co.uk (O.P.); k.d.do@amrc.co.uk (K.D.); a.molyneux@amrc.co.uk (A.M.); j.mcmanus@amrc.co.uk (J.M.); j.hughes@amrc.co.uk (J.H.)

**Keywords:** additive manufacturing, additive friction stir deposition, Al6061, process monitoring, temperature evolution, residual stresses

## Abstract

Additive friction stir deposition (AFSD) is a solid-state AM method that feeds, plasticizes, and deposits solid bars using frictional heat. Although the AFSD is a promising method, its limited technology readiness level precludes its wider use. The use of optimum process parameters is critical for achieving successful results, and closed-loop control of process parameters can improve quality even further by reacting to and resolving any unanticipated issues that arise during the process. This article investigates the utilization of a process monitoring setup including various sensors to examine temperatures, forces, vibrations, and sound during the AFSD of the Al6061 aluminum alloy. Furthermore, it benchmarks the outcomes of the same process’ parameter set with or without utilizing a proportional–integral–derivative (PID). Large thermal gradients were observed at various locations of the deposit. Significant fluctuations in temperature and force were demonstrated for the initial layers until stability was reached as the height of the deposit increased. It has been shown that the change in the process parameters may lead to undesired results and can alter the deposit shape. Finally, residual stresses were investigated using the contour measurement technique, which revealed compressive stresses at the core of the part and tensile stresses in the outer regions.

## 1. Introduction

The field of additive manufacturing (AM) has revolutionized the way materials are fabricated, offering unprecedented design freedom, material efficiency, and the ability to create complex geometries. Metal AM processes, such as laser powder bed fusion (PBF-LB) and directed energy deposition (DED), have demonstrated the potential to transform the manufacturing industry by enabling the rapid production of complex, customized parts without the need for dedicated dies or molds. Consequently, these processes have been employed across different sectors such as the aerospace sector, defense sector, and biomedical sector. However, significant constraints hinder the full acceptance of these additive manufacturing techniques in the industry, such as the requirement for a sealed chamber in LPBF, restricted build volume, and scalability issues [1]. In addition to the build volume, the building rates of PBF-LB and DED are lower than those of solid-state AM methods [2]. While the building rates for PBF-LB are 0.1 kg/h and 1 kg/h for DED, the building rates for solid-state AM methods can reach up to 10 kg/h for certain materials [1]. The energy consumption of solid-state AM methods has also been highlighted to be lower than powder-based methods like DED [3]. Finally, the cost of powder has been emphasized by previous researchers, as it leads to high expenses when DED or PBF-LB are used [3]. In this regard, the productivity and scalability of these processes have remained areas for improvement. On the technical side of things, powder-based AM methods pose issues such as the formation of high residual stresses leading to undesired deformations, anisotropic mechanical properties due to the columnar grain structure, and the need for subsequent processing [4,5]. Moreover, working with some materials is more difficult due to thermal properties or reactivity. For example, aluminum alloys that possess high reflectivity, thus low energy absorption, oxygen affinity, as well as susceptibility to cracking with high energy inputs, can pose even greater difficulties when using these techniques based on melting and solidification [6].

Solid-state AM methods, such as additive friction stir deposition (AFSD) or Friction Stir Additive Manufacturing (FSAM), have surfaced as a viable option in comparison to conventional fusion-based techniques [7]. AFSD leverages the principle of traditional friction stir welding to enable the deposition of metallic materials in a layer-by-layer fashion. The AFSD process, which stirs a square or cylindrical feedstock bar with a rotating tool, also known as a hollow shoulder, employs an actuator to press the material onto the upper surface of the substrate or prior layers to generate frictional heat [8]. As the frictional heat raises the temperature of the contact area, the material’s yield strength decreases, and the plasticized feedstock begins to flow. Subsequently, the device begins its movement based on the G-codes of the computer numerical control (CNC) machine to bond the plasticized material onto the surface beneath. By the transverse motion of the shoulder in a layer, a single track is deposited, which is typically wider than 15–20 mm. The 3-dimensional (3D) component is then obtained upon finishing this cycle for all layers.

**Figure 1:**
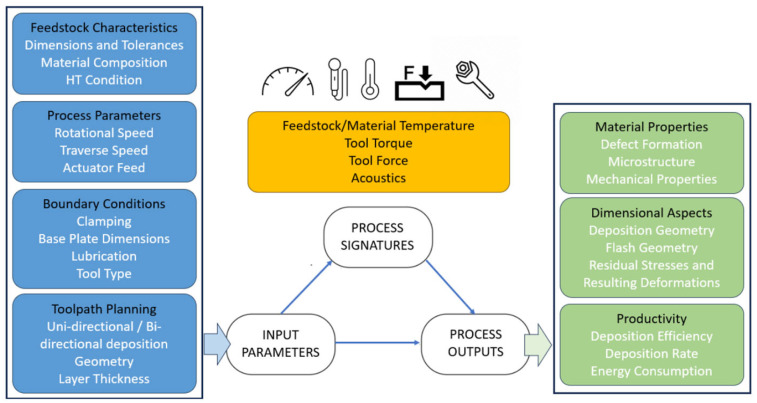
Input parameters, process signatures, and process outputs for AFSD by Yasa et al. (under CC BY 4.0 license) [7].

The AFSD process, a thermomechanical process, is affected by key process parameters, including layer height (mainly preset), spindle speed (also known as shoulder rotation frequency), actuator speed (also known as filler material feed rate), and traverse speed [9]. The layer height indicates the measurement between successive layers in millimeters (mm), whereas spindle speed refers to the number of rotations per minute (RPM). Conversely, actuator speed refers to the millimeters per minute (mm/min) progression of feedstock to the surface along the deposition (Z) axis, while traverse speed indicates the movement rate in a layer in mm/min. All these parameters can influence the process temperature and actuator forces. For example, Schmitz et al. [10] reported overheating with an increase in the spindle speed. Clearly, a rise in temperature can negatively affect the material, while an increase in force might cause the machine’s actuator to depreciate sooner than anticipated or the deposition to fail. A detailed study conducted by Kar et al. (2023) indicates a grain refinement occurring from the substrate to the deposited layer and emphasizes that the subsequent deposition influences the preceding layer, similar to a heat treatment [11]. Another point that was emphasized was the significant plastic deformation and its advantages for the grain structure [11]. A comprehensive understanding of these inputs, outputs, and process signatures is highly significant, as illustrated in Figure 1 [7].

Most of the defects, such as galling, beading, undesired local melting, or poor bonding leading to delamination in AFSD, stem from the construction of incorrect process windows [7]. While it is feasible to address these challenges via modeling and simulation techniques for more mature AM methods such as PBF-LB [12], there are only a limited number of modeling studies focused on the emerging AFSD process. The current state-of-the-art AFSD modeling studies utilize different approaches, as most of them try to predict process temperature. Some use heat source simplifications [13] to utilize available finite element method (FEM) software; others simulate the process fully by rotating the tool and calculating the frictional forces and subsequent temperature but are not computationally cost effective [14].

To enhance the process reliability of AFSD and enable proper construction of process windows that will lead to the right set of parameters for stable processing, monitoring and closed-loop control are crucial [15]. One of the pioneering studies for the monitoring of AFSD was performed by Zhang et al. [13]. In their research, aimed at validating Monte Carlo simulations, the temperature was measured in situ with infrared radiation imaging technology. During the AFSD of Al6061, they discovered the initial layer temperature exceeding 390 °C. Garcia et al. benchmarked the temperature profiles of the AFSD process for aluminum and copper alloys by considering exposure durations and reheating/cooling rates [16]. An infrared camera, along with embedded K-type thermocouples, was utilized to track temperatures, showing that the maximum values remain very near the melting point of the materials. They also revealed that the relationships between peak temperatures of aluminum and copper and process parameters varied [16]. Subsequent researchers, Yu et al., emphasized the necessity for additional details regarding material flow and proposed a technique to implement a tracer material in the feedstock to facilitate tracing the various stages of AFSD [17]. Merritt et al. aimed to tackle and reduce galling and beading by modifying the spindle speed to ensure that a thermocouple in the tool maintains a fixed temperature level [18]. In this manner, they were able to use a lower spindle speed compared to the speeds set by the original program. Finally, Chaudhary et al. used a pyrometer and a thermal imaging camera in a closed-loop system [19]. Additionally, they used an external heat source to decrease the thermal gradients.

The literature indicates that research on AFSD process monitoring is growing, although most of the studies utilize methods to monitor a specific process signature, such as temperature. This study contributes to existing scientific knowledge by simultaneously utilizing an embedded tool thermocouple, multiple thermocouples integrated in the substrate, as well as a thermal camera. While the thermal gradients at different locations of the build were examined with the help of various sensors, the consistency between contact and non-contact temperature measurement techniques was also verified. Besides temperature measurements, force, sound, and vibrations were monitored during the process, allowing for a correlation of all in the same environment and time scales. The impact of proportional–integral–derivative (PID) control on temperature and force for an optimized set of process parameters was also studied with an enhanced benchmark by incorporating an increased parameter set to monitor the evolution of temperatures. The correlations between temperature, force, sound, and vibrations were highlighted, especially for PID control. Residual stresses resulting from these process inputs and thermal gradients have also been investigated using the contour measurement technique.

## 2. Materials and Methods

All the experiments were performed on the MELD L3 machine located at the University of Sheffield Advanced Manufacturing Research Centre Northwest (AMRC NW). Because of its many uses, ranging from marine fittings to aerospace structures, Al6061 alloy with Al–Mg–Si alloying elements was selected as the base material [20,21]. The substrates were 400 × 76.2 × 25.4 mm^3^ blocks, and the deposition material was 500 × 12.7 × 12.7 mm^3^ square-sectioned bars from the same Al6061 alloy. Three sets of experiments were designed and conducted, along with process monitoring and data collection.

### 2.1. Specimen Design, Process Planning, and Execution

**Figure 2:**
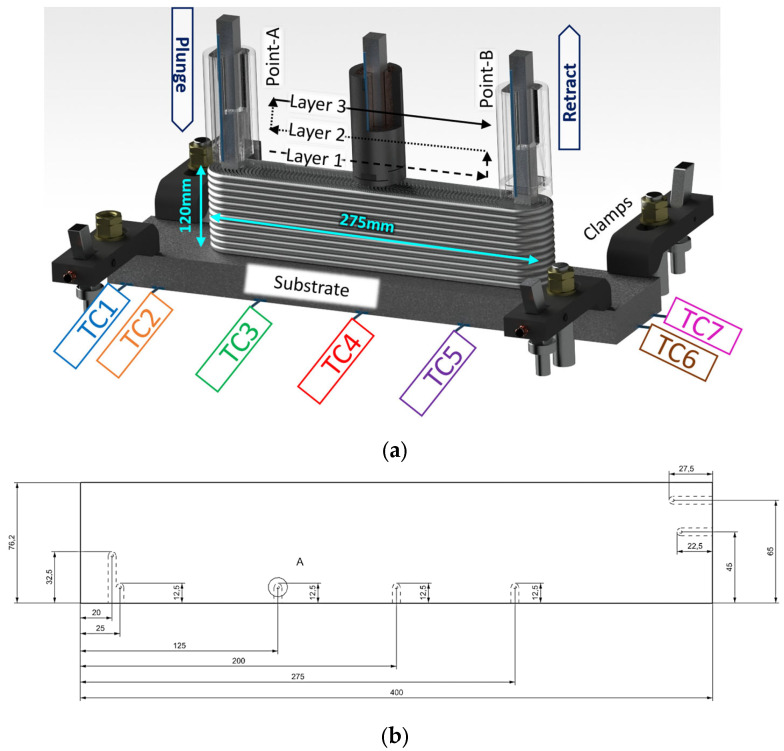
(**a**) Deposition strategy, specimen design, and thermocouple application, (**b**) thermocouple locations.

A longitudinal sample was designed to streamline thermal camera imaging from a single direction, with a line of sight of the build and tool. The specimen’s length was maximized without colliding with the side clamps, with its length fixed at 275 mm. The specimen’s width was mainly determined by the process parameters and the tool’s diameter, which was 38 mm. Finally, the specimen’s height was set to 120 mm to observe the thermal gradients along the Z axis. A zig-zag deposition strategy across layers was chosen to enable successive traverse motions in layers along the build direction. Each feedstock bar with a length of 500 mm was consumed after three layers. Holes were designed and drilled on the bottom surface of the substrate to fit external thermocouples, as shown in Figure 2, which illustrates the deposition strategy as well as the specimen design and exact locations of the thermocouples embedded in the substrate.

Maintaining a constant layer height of 2 mm across all experiments, three key process parameters were benchmarked during the process monitoring activities. As mentioned, these include spindle speed (rpm), indicating the rotations of the tool each minute, actuator speed (mm/min), representing how fast the feedstock is pushed by the actuator, and traverse speed (mm/min), which denotes the speed of tool movement in the X-Y plane. When PID is active, these parameters are adaptively controlled based on a thermocouple reading located within the tool. This control system is developed and implemented on the MELD L3 machine by the machine OEM [22]. This is especially critical for the first layer in the AFSD process to enable good bonding and to eliminate delamination. Depending on the phase of the deposition, the parameters are adaptively controlled as shown in Table 1 within Experiment-1 settings. The deposition start is considered the contact phase, followed by stabilization phase, and, finally, the traverse phase takes place to adjust the parameters at the beginning of table motion and the rest condition parameter status to be used for the rest of the zig-zag deposition pattern. Baseline parameters for each phase displayed in the first column of Table 1 (with Exp-1) were determined from previous experiences with parameters that typically yield optimal tensile properties at room temperature. Along with these, a proportional–integral–derivative controller (PID controller or three-term controller) has been employed for the baseline parameters. A PID controller is frequently used to operate machinery and processes that need constant control and automated adjustment [23]. It is frequently used in industrial control systems and other settings where ongoing control via modulation does not necessitate human involvement. Furthermore, it is one of the most common methods regarding manufacturing control [24]. Demonstrations of manufacturing process control with PID involve modifying the grinding speed, regulating the wire feed for metal arc welding, altering the laser power in PBF-LB, and adjusting the friction stir welding rotations to minimize temperature fluctuations [24]. The PID controller automatically compares the desired target value (setpoint or SP) with the actual value of the system (process variable or PV) for a specific target process signature. In this study, temperature was utilized as the set point for the tool thermocouple reading. The target temperature for the AFSD is set based on different factors. Fundamentally, the temperature value reached should be below the melting point of the feedstock material, as this is a solid-state process [25]. Still, the maintained temperature should be high enough to facilitate the plastic deformation and flow of the material. Although the research on the AFSD is still emerging, the temperature values from conventional processes like forging, which have high deformation and material flow, might shed light on the process. At a temperature of 480 °C and a strain rate of 10 s^−1^, flow stresses for Al6061 have been reported to stay below 50 MPa, within the range of 10–60% strain [26]. Additional research has examined Al6063 and shown a decrease in forming force to 10 kN at temperatures exceeding 450 °C [27]. In a unique study that conducts AFSD on Al6061 and provides comprehensive details, PID set temperature values of 400 °C, 420 °C, and 440 °C were utilized according to the operators’ estimated settling [18]. This article employs a PID set temperature of 470 °C, situated between the AFSD and forging temperature range for Al6061 materials, based on the prior literature. To examine the effect of PID on the results, Experiment-2 (Exp-2) was planned and carried out with parameters akin to Exp-1 but without the PID control. Since the PID was deactivated, some initial parameters like contact and stabilization were adjusted to avoid any job failure based on previous experience. Finally, Experiment-3 (Exp-3) was intentionally conducted with an increased spindle speed to cause overheating and observe the potential outcome (see Table 1 for the parameter sets).

### 2.2. Process Monitoring and Data Collection

**Figure 3:**
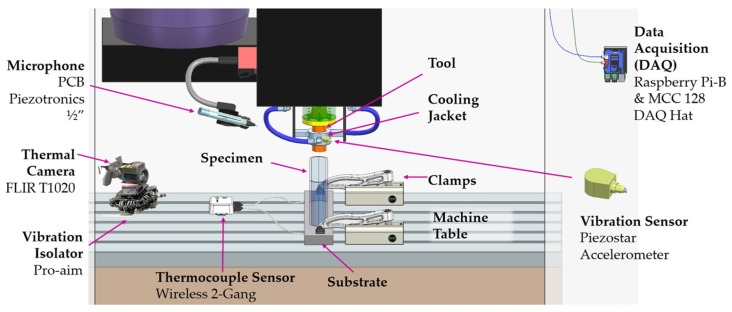
AFSD process monitoring and data collection architecture.

For the first time in the literature, a process monitoring and data collection system has been established for this process to simultaneously capture multiple process signatures such as temperature, force, sound, and vibrations, as illustrated in Figure 3.

#### 2.2.1. Temperature Monitoring

Temperature evolution during the deposition was traced by utilizing two different methods from various locations. An OEM-embedded K-type thermocouple was positioned near the tool’s tip to record temperature versus time via the machine’s user interface in comma-separated value (csv) file format, with the aim being to trace the temperature evolution in the tool body. To be able to capture the thermal gradients on that part, additional K-type thermocouples were embedded in the substrate at 7 locations (see Figure 2). The RS PRO 787-7778 thermocouples, which can measure temperatures reaching up to 750 °C, were linked to a laptop workstation through a Picologger TC-08 (Pico Technology, St. Neots, UK). Finally, a FLIR Model T1020 thermal camera was installed on the machine table with the help of the Proaim Pro Camera Vibration Isolator (Chandigarh, India) with a custom 3D printed adapter. The emissivity, environmental temperature, and atmospheric humidity were set to 0.6, 20 °C, and 50%. Among these values, the emissivity value has been chosen from a variety of values reported in the literature for different welding techniques, since there is no emissivity value in the literature concerning the AFSD [28]. Decoding and temperature extraction were performed with custom codes using FLIR’s software development kit version 2024.5.0. The tool tip temperature was calculated by taking the location and temperature of the hottest part of each frame.

#### 2.2.2. Forces

The feedstock actuator’s function is to push the feedstock bar into the spindle and through the tool. The actuator can push bars with up to 26.7 kN of force at speeds up to 508 mm/min. Tracking the axial loading is essential, as persistent and prolonged use of high forces can result in mechanical issues occurring sooner than expected. The estimated actuator force is determined using the drive feedback current and the motor torque constant. Actuator force calibration was performed by the machine OEM during routine maintenance. The actuator force (N) was determined by multiplying the drive torque (N.m) by 9890 and the efficiency factor, which was determined by positioning a load cell beneath the actuator and correlating its output as 73%. Similarly to the embedded thermocouple temperature data collection, the axial force was also collected via the machine’s user interface in csv file format.

#### 2.2.3. Sound and Vibrations

While shock and vibration were measured through a Piezostar accelerometer (Kistler Group, Winterthur, Switzerland) that was mounted on the tool cooling jacket as shown in Figure 3, the sound was collected via a PCB Piezotronics ½”prepolarized free-field microphone. Both were connected via coaxial to an MCC172 DAQ Hat attachment for the Raspberry Pi. Integrated Electronics Piezo-Electric (IEPE) protocols were followed with a sampling rate of 12,800 Hz. Raw voltage values were read directly from the files. SciPy signal library spectrogram was used to calculate consecutive Fourier transforms with an alpha value of 0.25, calculating the power spectral density. The densities were extracted and plotted at 10*log(v), where v is the density of a given frequency at a given time point. The acceleration was calculated by dividing the signal of the accelerometer by the sensitivity of the device (20 mV/g). Data were plotted using matplotlib’s pcolormesh for each slice of time.

### 2.3. Surface Topography and Residual Stresses

In order to correlate the obtained process monitoring data with the part quality, the obtained surfaces were characterized by optical microscopy (Keyence VHX microscope) for surface characteristics and defect analysis. Three separate regions on the top surfaces of samples have been photographed at 20× magnification. Secondly, residual stresses within the produced parts were measured and evaluated. Residual stresses are prevalent in most of the fusion-based AM processes because of the rapid heating and cooling cycles involved during the layer-by-layer build process, creating significant temperature gradients within the part, which lead to differential contraction and, ultimately, internal stresses that remain even after the deposition is complete. Although residual stresses are less dominant in the AFSD process, they can still be challenging depending on the deposited volume and deposition strategy. Thus, in this study, the contour method was chosen for measuring residual stresses [29]. The contour method to assess residual stresses utilized an Agie Charmilles CUT P-550 wire electric discharge machining (WEDM) to cut Exp-1 and Exp-2 specimens into two halves along the mid-longitudinal plane, which led to deformation of the newly revealed surfaces as a result of the release of internal stresses. These deformations, or “contours”, are then precisely measured on a Renishaw Agility coordinate measuring machine (CMM) and used to calculate the original residual stress distribution within the material using a finite element analysis, essentially “reversing” the deformation to determine the stresses that caused it. The data obtained from CMM was analyzed using Python software version 3.12 to process the data, including elimination of the noise, averaging two surface outputs, smoothing out, reversing, and exporting them in a tabular format compatible with Ansys. The tabulated data were subsequently imported into Ansys R2024 and designated as displacement boundary conditions for the half geometry of the specimens. With the material data for Al6061 sourced from the Ansys library, residual stresses were computed by fixing the remote planar surfaces of the specimen geometries.

## 3. Results and Discussions

### 3.1. Temperature Evolution

**Figure 4:**
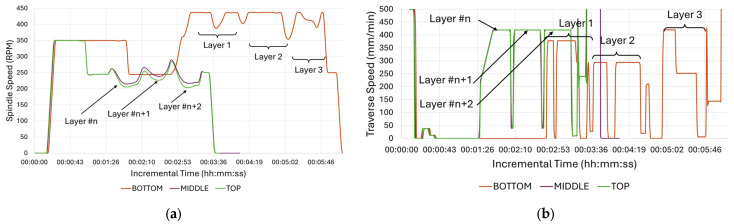
Process parameter monitoring for three positions (bottom, mid-level, and top) and three layers at each position: (**a**) spindle speed (rpm) and (**b**) traverse feed (mm/min).

As shown in Table 1, Exp-1 was carried out with the PID controller turned on and optimal baseline parameters. The influence of PID control on the process parameters is demonstrated in Figure 4. The alterations in spindle speed (rpm) and traverse speed (mm/min) managed by the PID controller are benchmarked for three positions: bottom, mid-level, and top of the build. For every position, the duration of a single feedstock was recorded, which corresponds to three layers, as previously detailed in the zig-zag deposition method. As can be seen in Figure 4, the PID adjusts the spindle speed and traverse speed based on the set value. The spindle speed required almost three minutes to reach its deposition value at the bottom position, whereas it only took less than a minute for the middle and top positions. The same can be noted for the traverse speed despite higher variations.

The delay and offset may be linked to the proximity of the tool thermocouple to the substrate mechanically fixed on the machine bed. At the bottom of the build, as the tool thermocouple is positioned much nearer to the machine bed, due to the higher heat absorption through the machine bed, which is a consequence of its substantial volume and mass, the temperature increased slowly. In the middle and top sections of the build, the heat conduction can only occur through the cross-section of the build, allowing a faster increase in the temperature measured by the tool thermocouple. While the process parameters for all experiments have been recorded via csv file results, those for Exp-2 and Exp-3 are not detailed in graphs since they adhered strictly to the CNC program with fixed parameters as given in Table 1.

**Figure 5:**
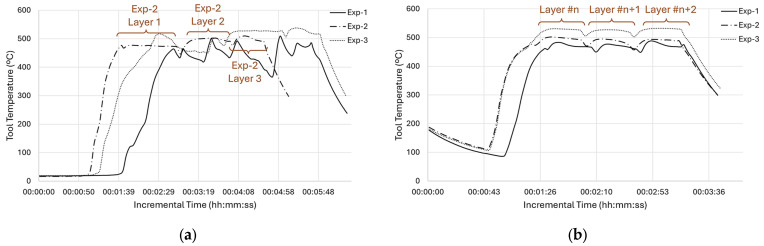
Temperature profiles for all experiments obtained from the tool thermocouple: (**a**) bottom and (**b**) top positions of the build.

The temperature changes for all experiments were evaluated according to the readings obtained from the tool thermocouple, as demonstrated in Figure 5, at both the bottom and top of the builds. The figure clearly shows that the PID controller causes an initial delay before reaching the set temperature of 470 °C, both for the top and bottom positions. The temperature variations necessary to maintain the average temperature at the set value are more noticeable at the bottom than the case at the top. This finding is in line with the process parameters, indicating a higher fluctuation in the spindle speed and traverse speed at the bottom, as depicted in Figure 4. Besides the bottom position results of Exp-1, the remaining trials reflect the deposition strategy and exhibit three humps and two valleys that align with the three zig-zag layer depositions. Exp-3, which was deliberately designed and set to higher spindle speeds, exhibited average temperature readings exceeding 500 °C, while Exp-2, configured with parameters akin to Exp-1, but with no PID control, displayed average temperature values ranging from 470 to 500 °C. This finding is in good agreement with the previous literature. Williams et al. (2023) performed AFSD trials on Al7020 with three different parameter sets and reported the highest temperature in the case where the tool rotation speed increased [30].

**Figure 6:**
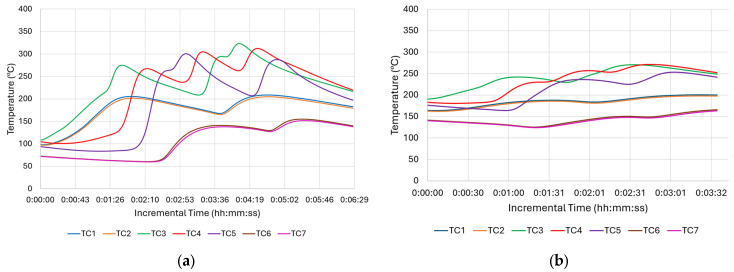
Temperature profiles of Exp-1 obtained from substrate thermocouples: (**a**) bottom and (**b**) middle positions.

While the tool thermocouple readings provided valuable information about the thermal state of the process at the hottest point of the process, to evaluate the thermal gradients, more information is required from locations closer to the deposition. In this regard, data from the additional thermocouples placed at the substrate’s base were taken into account, as shown in Figure 6.

As seen in Figure 6a, the initial plunging, or engagement, takes time until it reaches the tool’s PID set point. When the tool thermocouple reaches 470 °C, TC3 reaches approximately 300 °C. Although the highest temperature peaks that are between 300 and 350 °C come from TC3, TC4, and TC5, which are closer to the deposition area than the others, they do not reach the PID set temperature, 470 °C. This is measured through the thermocouple inside the tool and corresponds to almost 80% of the melting temperature for this material. This significant finding shows the level of thermal gradient between the substrate and the deposition area close to the tool. Another important thermal gradient was experienced longitudinally. While TC3 had its valley at Point-B or at the end of the first traverse move, TC5 reached its peak at the very same time. On the other hand, the TC4 thermocouple, which was between TC3 and TC5, had its peaks just after TC3 and TC5 with some offset due to bidirectional zig-zag motion. Stubblefield et al. (2021) demonstrated similar trends along traverse direction for an AFSD simulation study on Al6061 alloy [31]. Offsets were observed among the temperature peaks of the advancing side, middle side, and re-treating side [31]. Nonetheless, the magnitude of the thermal gradients in their research was reduced because they utilized a very thin substrate (6.35 mm), resulting in faster overall heating. Among the corner thermocouples, TC1 and TC2, being close to the long edge, showed slightly higher peaks than TC6 and TC7, which were located on the short edge. This is expected due to their position on the periphery (Figure 6a). The peak times of TC1 and TC2 were identical to TC3. Still, the magnitude of their peaks at approximately 200 °C was significantly lower than the maximum temperature reached with TC3. When the maximum tool temperature of 470 °C is considered, the extreme thermal gradient for the whole structure can be appreciated. As the height of the deposit increases, the substrate thermocouples’ distance with the friction area increases too. Thus, the maximum temperature sensed decreased to 250 °C (Figure 6b), where it was above 300 °C (Figure 6a) at the beginning of deposition. This difference is mainly attributed to the loss of heat due to thermal conduction. As the max temperature values decreased, the difference between the start of each layer and its peak became smaller. This difference is even negligible at the top of the deposition. Exp-2, which uses the same parameters without a PID control, showed similar gradient variations.

**Figure 7:**
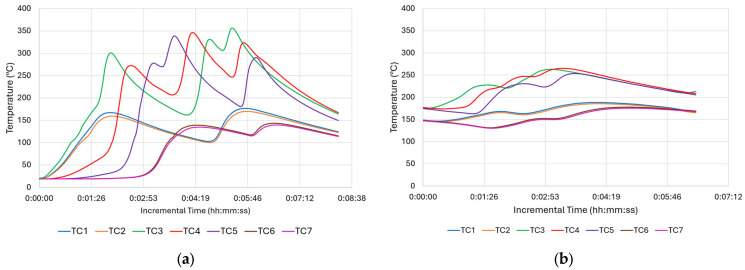
Temperature profile of Exp-3 obtained from substrate thermocouples: (**a**) bottom and (**b**) mid-level positions of the build.

Figure 7 shows the temperature evolution of Exp-3 for the bottom position. As Exp-3 utilized a higher spindle speed, which had an impact on the frictional force and thus the generated heat, its deposition peaks at the bottom were over 350 °C, as shown in Figure 7a, whereas these values were only over 300 °C for Exp-1 (Figure 6a). Another expected result is the time interval between each peak. Since Exp-3 utilized less traverse speed than Exp-1, the layer times were longer and thus showed longer intervals between each layer. As can be seen in Figure 7b, the peak temperature of each layer was less than the peak temperatures that were collected at the bottom of the build.

**Figure 8:**
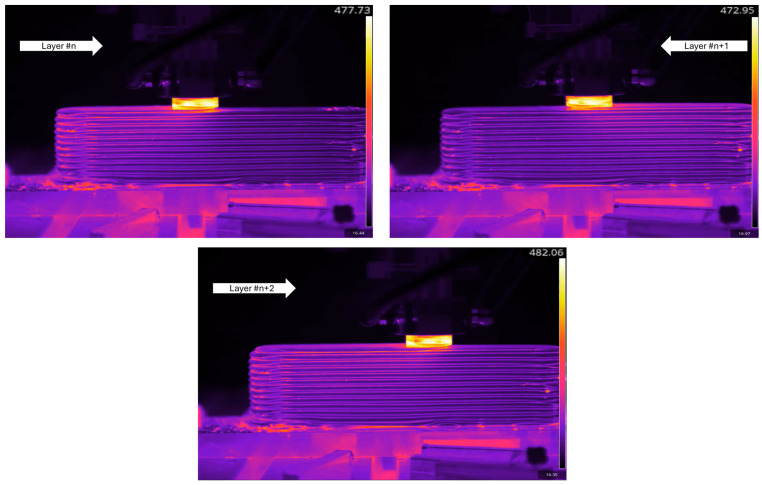
Thermal camera images of three successive layers during the medium height deposition of Exp-1.

Using the thermocouples integrated in the tool and substrate, the temperature evolution as well as the thermal gradients could be evaluated. Depending on the final design of the part, it is possible that the substrate can become a part of the final component, and this necessitates the implementation of a non-destructive process. This research also showcases the utilization of a thermal camera to evaluate its monitoring capabilities and to compare the derived readings with traditional contact-based temperature assessments. As outlined in the Materials and Methods section, the FLIR Model T1020 thermal camera was mounted on the machine table using a Proaim Pro Camera Vibration Isolator, and the videos were recorded according to the specified settings. The required stability was accomplished through the setup, allowing for the recording of vibration-free videos. Figure 8 displays some screenshots of thermal camera imaging for Exp-1 at middle height. As seen, sequential traverse movements in accordance with the zig-zag strategy show temperature values ranging from 472 to 482 °C. While these relate to the external surface of the tool and the tool-deposit interface, the measured values align well with the thermocouple embedded within the tool. This agreement is not restricted to this instance but applies to all experiments. Some unrelated hotspots near the substrate were noticed, and this is mainly believed to be reflectivity of the substrate’s side surfaces.

Another notable finding is the prominence of immediate thermal gradients. As the tool moves in the longitudinal direction of the component at every layer, a red emission area that indicates a temperature range of 245–265 °C trails behind the tool. Nonetheless, this region of red emission persists only for a distance that is equal to or slightly greater than the tool’s diameter at the deposition surface. It gradually vanishes through earlier layers, forming a subtler diagonal shape. Conversely, the remaining section shows black-purple emissions that correspond to a temperature range of 95–105 °C.

### 3.2. Axial Force Measurements

**Figure 9:**
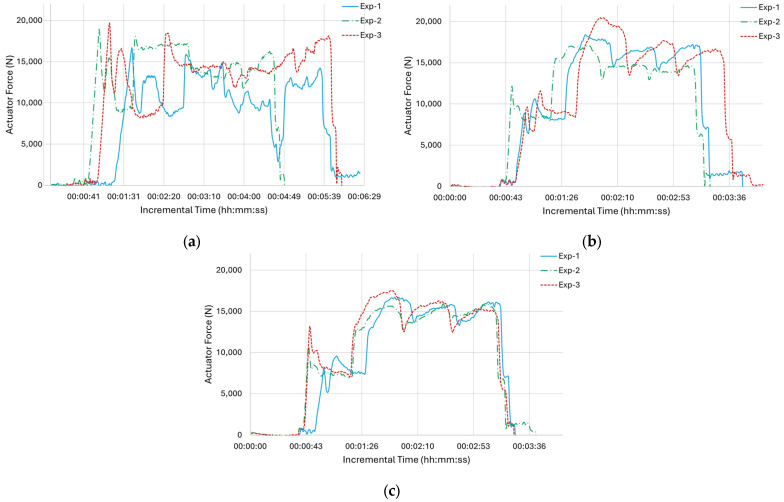
Force profile for all experiments obtained from the tool thermocouple: (**a**) bottom, (**b**) middle, and (**c**) top.

The axial forces calculated from the drive feedback current and motor torque constant, considering a predetermined actuator efficiency of 73%, were saved as a CSV file, and their values in N were graphically represented over time (Figure 9). In nearly every experiment and at every site, the forces remained under 20 kN, with the sole exception being Exp-3 at middle height. However, at middle height, the maximum of Exp-3 was only 390 N higher than the 20 kN, which is negligible. All experiments showed higher peaks when beginning the deposition at the bottom. This can be linked to the ambient specimen temperature in the range of 20–25 °C initially, reaching at least 150 °C in all specimen locations as the deposition went on. This resulted in reaching even higher temperatures for the consecutive layers, as illustrated in Figure 8. Though this temperature may not be the precise forging temperature for Al6061, previous research clearly indicates that the yield strength of Al6061 begins to significantly decrease within the range of 250–300 °C [32]. Another common finding from the three experiments was the significant force fluctuations observed at the bottom of the deposition. Ghadimi et al. (2024) demonstrated trends alike while mapping the forces for different layer thicknesses and process parameters of the same alloy [33]. In their research, they documented smaller fluctuations in forces from the start to the end of the first layer compared to the following layers. They also noted that the material of the workpiece becomes more malleable as the temperature of the feedstock rises [33]. This situation did not persist for the middle and top areas, and three notable humps along with two valleys were noted, resembling the temperature recorded from the tool thermocouple. Similarly, to the temperature trends, the force increases in Exp-1 began with a delay of half to one minute compared to the other two experiments.

### 3.3. Sound and Vibration Observations

**Figure 10:**
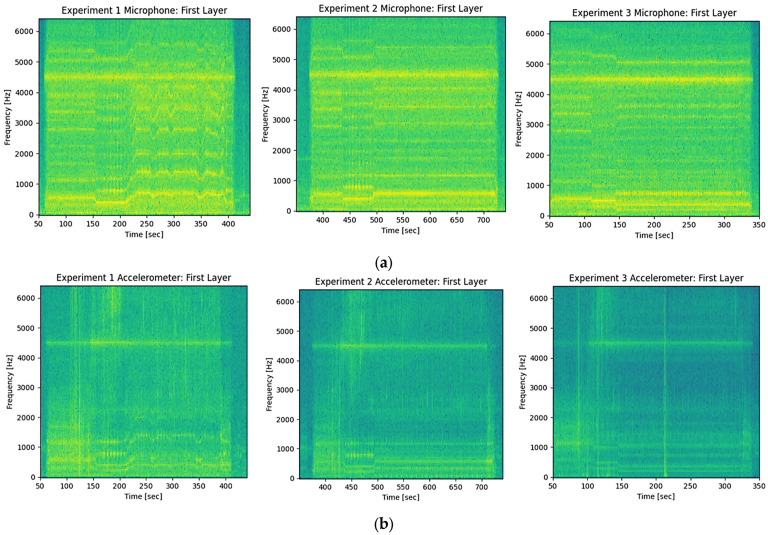
Recordings from all experiments at the first layer (**a**) acoustic sensor and (**b**) accelerometer for vibration monitoring.

The heat maps given in Figure 10 for sound and vibrations were created utilizing an absolute time scale ranging from 0 to 450 s for the bottom of the deposition. The horizontal axes of the graphs indicate time, while the vertical axes illustrate the frequency. The heat maps displaying colors from yellow to green also provide a relative indication of the voltage measured during the experiments, as shown in Figure 10.

As evident from the microphone and accelerometer readings, a prominent frequency around 4500 Hz is observed and associated with the machine body having a substantial mass. This can be observed with all parameter settings. The only major difference observed between the three sets of process parameters is the fluctuation in frequency and voltage observed in Exp-1. This is mainly due to the PID adjusting the spindle speed, and it was observed in the temperature and force readings presented earlier. The acoustic signal can also be perceived with the human ear throughout the process. The distinct noise begins once the spindle motor starts to rotate, and only minor alterations can be detected as the feedstock penetrates the substrate. As Exp-2 and Exp-3 primarily change the spindle speed based on the CNC program, frequency variations are significant only during those specific moments. For instance, Exp-2 begins its initial layer at approximately 400 s and undergoes a frequency shift around 450 s. This pertains to changes in the starting spindle speed during traversal (see Table 1) and can be observed in temperature or force graphs in Figure 5a or Figure 9a, where the ramp is evident between the 40–50 s incremental time interval. In this context, it can be said that the base frequency originates from the machine, while the dominant process frequency arises from the spindle motor’s rotational speed. However, further investigation is necessary on how to utilize these sensors for detecting stable or unstable processing.

### 3.4. Surface Characteristics and Residual Stresses

**Figure 11:**
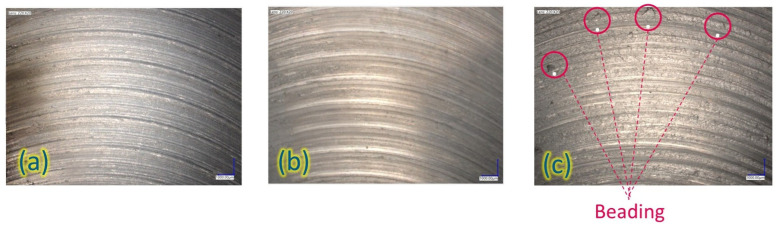
Upper surface topographies of specimens: (**a**) Exp-1, (**b**) Exp-2, and (**c**) Exp-3.

Like chevrons in laser powder bed fusion or fish scales in welding, onion skin is a characteristic of the AFSD process [7,34]. The upper surfaces of all samples in this study displayed the onion skin structure, as shown in Figure 11. The pitch of the onion skin pattern is theoretically determined to match the advancement per revolution of the tool, indicating that it results from the interaction between the deposited material and the tool’s edge as it rotates [34]. The measurements for the pitch values were performed at three different locations with three repetitions. As shown in Table 2, the ring diameters across all experiments were similar, ranging from a minimum of 34.9 mm to a maximum of 38.2 mm. This result was expected since the same tool has been used for all experiments, and it is the main influence on the ring diameter.

Conversely, the pitch distances varied significantly. It was observed that the upper surface of the Exp-1 specimen exhibited variable pitch distances as a result of the use of the PID controller on process variables. Exp-2 and Exp-3, in which PID was not activated, demonstrated constant pitch distances at various locations. The pitch distance of Exp-3 was lower as its spindle speed was set to a higher level. The rise in spindle speed for Exp-3 resulted in additional rotations for the same traverse distance, causing overheating. Moreover, Exp-3 exhibited uneven and incomplete rings with some drop-like formations that were scattered randomly (see Figure 11c). This finding is in line with previous literature, as Merritt et al. (2022) reported similar outcomes, named them as “beading”, which was mainly attributed to the overheating [18].

**Figure 12:**
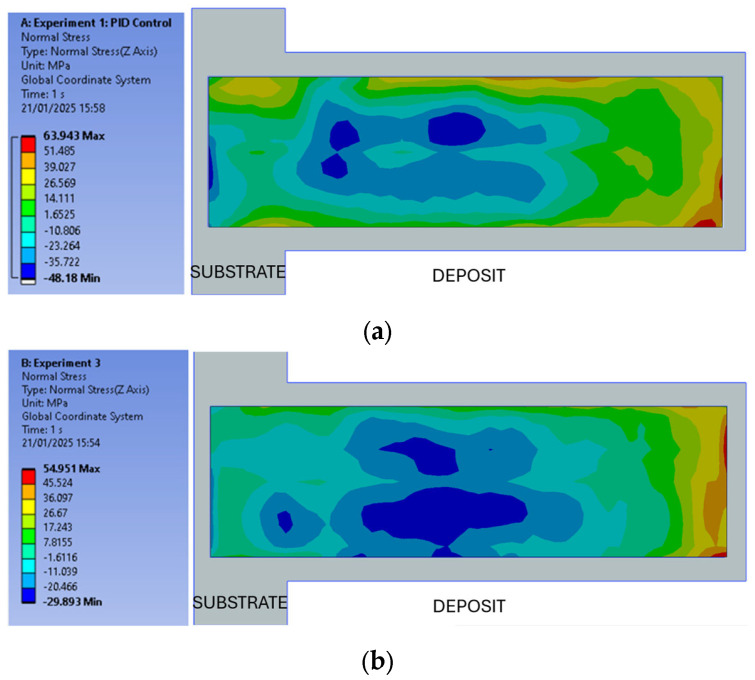
Residual stress measurement results: (**a**) Exp-1 and (**b**) Exp-2.

Since the internal residual stresses of materials subjected to thermal processing typically vary with temperature fluctuations, the stress states of the samples were assessed. The goal was to compare the stress states of two specimens that utilize similar process parameter sets, one employing PID control and the other not. In this respect, Figure 12 shows the benchmark between Exp-1 and Exp-2 specimens. Both Exp-1 and Exp-2 gave compressive stresses at the core and bottom of the deposits, and tensile stresses around them. The maximum tensile stresses were experienced near the top. The max stresses at the top can be explained by the fast cooling of the top layer with the help of ambient convection. Exp-2 (Figure 12b) gave slightly lower compressive and tensile stresses compared to Exp-1 (Figure 12a). The tensile stress value decreased from 64 MPa to 55 MPa, and the compressive stress value decreased from −48 MPa to 30 MPa. Though there is no equivalent study in the literature that employs the same geometry, same materials, identical process parameters, and the same measurement method, there is one investigation by Yakubov et al. (2024) [35] that can be referenced. In their study, they processed the same Al6061 alloy using a 300 rpm spindle speed and 152 mm/min actuator feed. The 2D graphs resulting from neutron diffraction analyses in the longitudinal direction indicated a stress exceeding 50 MPa at a deposit height of 5 mm [35]. Like this article, the stress orientation was predominantly compressive for the substrate. From the residual stress measurements, it can be concluded that, provided that the parameters are correctly selected and kept constant, no significant difference is observed in the direction of developed residual stresses with or without PID, although the axially obtained profile loadings are distinct.

## 4. Conclusions

In this study, a thorough evaluation of the AFSD process was carried out for three different process parameter sets using a process monitoring system that included a variety of sensors to detect process signatures of the AFSD process. Temperature, being the key variable related to the process and residual stresses, was tracked using three different sensors: a tool thermocouple, embedded thermocouples in the substrate, and a thermal camera. All temperature monitoring techniques showed consistent results. Thermal gradients were observed between the tool and the substrate corner, ranging from 470 °C to 150 °C. The axial force was monitored by detecting the current through the machine drive. Regardless of the process parameter or PID employed, the force fluctuations were greater at the bottom of the deposition and decreased towards the top. The recordings at higher build positions exhibited more uniform force graphs, and the layer transitions were significantly more evident. Nonetheless, Exp-1 utilizing the PID exhibited greater fluctuations compared to Exp-2 and Exp-3. All measurements of sound and vibration exhibited their maximum values at a frequency range between 4000 and 5000 Hz. In addition to the dominant frequency range, Exp-1 exhibited variations throughout the entire build as it adjusted the spindle speed using PID control. More assessments and targeted vibration research are needed to determine the best use of these sensors to detect stable or unstable processing. The contour residual stress technique was employed at the center of Exp-1 and Exp-2 to analyze the residual stresses and to evaluate the effect of PID control under comparable process parameters. Residual stresses ranged from −48 MPa in compression for the inner area of the component to 63 MPa in tension for the outer area of the component. The tendency to tensile stresses in the outer region can be attributed to exposure to greater temperature gradients during the cooling process of the part, caused by the convection of surrounding air. As the component is secured with clamps, the internal area tends to balance out, resulting in compressive stresses.

## Figures and Tables

**Table 1:** Process parameters used in the conducted experiments.

Parameter	Status	Exp-1 (PID)	Exp-2 (No PID)	Exp-3 (No PID)
Spindle Speed (rpm)	Contact	350	350	350
	Stabilization	245	310	245
	Traverse Start	Variable around 437	245	350
	Traverse Rest	Variable around 245	245	350
Actuator Speed (mm/min)	Contact	20	20	35
	Stabilization	25	25	20
	Traverse Start	Variable around 120	125	120
	Traverse Rest	Variable around 72	125	100
Traverse Speed (mm/min)	Contact	0	0	0
	Stabilization	0	0	0
	Traverse Start	Ramping variable around 378	195	195
	Traverse Rest	Variable around 420	420	385

**Table 2:** Ring diameters and pitch distances between consecutive rings.

Experiment	Location at the Top of the Build	Ring Diameter (mm)	Pitch Distance (µm)
Exp-1 (PID)	1	34.9 ± 2.2	1263 ± 205
	2	36.9 ± 1.5	725 ± 176
	3	35.9 ± 1.3	921 ± 66
Exp-2 (No PID)	1	34.8 ± 1.5	1511 ± 43
	2	38.4 ± 1.6	1547 ± 63
	3	37.0 ± 1.4	1504 ± 98
Exp-3 (No PID)	1	37.7 ± 1.3	762 ± 33
	2	38.2 ± 1.4	753 ± 49
	3	37.0 ± 1.2	786 ± 57

## Data Availability

The original contributions presented in this study are included in the article. Further inquiries can be directed to the corresponding author(s).

## Abbreviations

The following abbreviations are used in this manuscript:

3D	Three Dimensional
AFSD	Additive Friction Stir Deposition
AM	Additive Manufacturing
AMRC NW	Advanced Manufacturing Research Centre Northwest
CMM	Coordinate Measuring Machine
CNC	Computer Numerical Control
CSV	Comma-Separated Value
DED	Directed Energy Deposition
EXP	Experiment
FSAM	Friction Stir Additive Manufacturing
IEPE	Integrated Electronics Piezo-Electric
MIN	Minute
RPM	Rotations per Minute
PBF-LB	Laser-Based Powder Bed Fusion
PID	Proportional–Integral–Derivative
WEDM	Wire Electric Discharge Machining
